# Effect of iRoot Fast Set root repair material on the proliferation, migration and differentiation of human dental pulp stem cells *in vitro*

**DOI:** 10.1371/journal.pone.0186848

**Published:** 2017-10-23

**Authors:** Yan Sun, Tao Luo, Ya Shen, Markus Haapasalo, Ling Zou, Jun Liu

**Affiliations:** 1 State Key Laboratory of Oral Diseases, Department of Endodontics, West China Hospital of Stomatology, Sichuan University, Chengdu, China; 2 Division of Endodontics, Department of Oral Biological and Medical Sciences, Faculty of Dentistry, University of British Columbia, Vancouver, British Columbia, Canada; 3 State Key Laboratory of Oral Diseases, Department of Orthodontics, West China Hospital of Stomatology, Sichuan University, Chengdu, China; Università degli Studi della Campania "Luigi Vanvitelli", ITALY

## Abstract

The present study investigated the effect of iRoot Fast Set root repair material (iRoot FS) on the proliferation, migration and differentiation of human dental pulp stem cells (hDPSCs). The hDPSCs were treated with eluates of iRoot FS at concentrations of 0.2 and 2 mg/mL, referred to as FS0.2 and FS2, respectively, and Biodentine (BD; Septodont, Saint Maur des Faussés, France) eluates at the corresponding concentrations as positive controls. A CCK8 assay was performed to determine cell proliferation. Wound healing and transwell assays were conducted to examine cell migration. Osteogenic differentiation was evaluated based on alkaline phosphatase activity, Alizarin Red S staining and quantitative real-time reverse-transcriptase polymerase chain reaction (qRT-PCR) to analyze the mRNA expression of differentiation gene markers. Cell proliferation was higher in the FS and BD groups than in the blank controls at 3 and 7 days. Moreover, FS0.2 enhanced cell migration and significantly promoted the osteogenic differentiation of hDPSCs. These findings suggested that iRoot FS is a bioactive material that promotes the proliferation, migration and osteogenic differentiation of hDPSCs.

## Introduction

Dental pulp exposure commonly results from caries and trauma. Direct pulp capping (DPC) is the most suitable treatment for this condition. The medicament or material is placed on the exposed pulp to maintain pulp vitality and induce the formation of reparative dentin [1]. The pulp-capping material is an important factor influencing the survival of the pulp [2]. An ideal pulp-capping agent should be easy to use, provide a good seal to dentin, cause little or no irritation to the pulp tissue, promote cell attachment and migration, and stimulate the production of reparative dentin.

Mineral trioxide aggregate (MTA) was introduced for pulp capping because calcium hydroxide has no inherent adhesive properties and provides a poor seal, and the self-cure formulations are subject to dissolution over time [3–8]. However, reflecting the long setting time, discoloration of tooth structure and gingival tissue, presence of toxic elements in the material composition, and difficulties handling and removing this compound, MTA shows disadvantages as a pulp-capping material [9–11]. Thus, the ideal pulp-capping agent remains elusive. Biodentine is a novel biomaterial designed for dentin replacement, which has a better performance compared with MTA [12], including the setting time, handling properties and biocompatibility. Biodentine has been well studied and widely applied for clinical use [13–15]. Therefore, Biodentine is an ideal positive control for pulp-capping material, as demonstrated in the present study.

The iRoot materials (Innovative BioCeramix, Vancouver, BC, Canada), including iRoot SP, iRoot BP, and iRoot BP Plus, are calcium silicate-based bioceramic materials used as non-shrinking, insoluble, aluminum-free, and favorable biocompatible dentin substitutes [16–19]. iRoot Fast Set root repair material (iRoot FS) is a modification of iRoot Putty Plus. This modified material is biocompatible, non-mutagenic, non-allergic and well tolerated by subcutaneous tissue. Recent studies have demonstrated that iRoot FS has a faster setting time (within an hour) and hydrating process than MTA, ERRM Putty (Brasseler USA, Savannah, GA, USA), and IRM (Dentsply Caulk, Milford, DE, USA). iRoot FS also has a similar apical sealing ability as MTA [20,21]. Despite these reported advantages, only two studies have investigated the biological performance of iRoot FS [22,23]. Jiang *et al*. [22] compared the cytocompatibility of iRoot BP Plus, iRoot FS, ProRoot MTA, and Super-EBA using osteoblast-like cells (MG63) and mouse connective tissue fibroblasts (L929). Among these materials, iRoot FS showed the best performance in promoting cell adhesion. Qiao *et al*. [23] reported that iRoot displayed good mineralization capabilities *in vitro* and promoted the differentiation and mineralization of MG63 cells. There is currently no information concerning *in vitro* pulp cellular responses to iRoot FS as a pulp-capping material. The regeneration of the damaged pulp-dentin complex, for which the pulp-capping materials are primarily used, depends on the migration, proliferation and differentiation of hDPSCs [24]. The objective of the present study was to evaluate the effect of iRoot FS on the proliferation, migration, and differentiation of human dental pulp stem cells (hDPSCs).

## Materials and methods

### Material preparation

The eluates of Biodentine (BD; Septodont, Saint Maur des Faussés, France) and iRoot FS (FS; Innovative Bioceramix, Vancouver, BC, Canada) were prepared at two concentrations: 0.2 and 2 mg/mL, referred to as BD0.2, BD2, FS0.2 and FS2, respectively. Briefly, BD and FS were premixed in paste form according to the manufacturer’s instructions under sterile conditions and subsequently set for 24 hours at 37°C. The solidified paste was ground into a powder and subsequently dissolved in Dulbecco’s modified Eagle’s medium (DMEM, HyClone, Thermo Fisher Scientific, Waltham, MA, USA) at a concentration of 20 mg/mL. The solution was vortexed and incubated at 37°C with 100% humidity for 3 days, followed by filtration and dilution to the appropriate final concentrations as complete media supplemented with 10% fetal bovine serum for use [25].

### Cell culture

This study was approved through the Institutional Review Board of West China Hospital of Stomatology, Sichuan University (WCHSIRB-D-2015-087). The hDPSCs were harvested and cultured as previously described [26]. Briefly, the premolars extracted from 4 adolescents (12 to 14 years old) for orthodontic reasons were collected after obtaining informed consent. The dental pulps were isolated, cut into small pieces (1 × 1 × 1 mm) and digested in a solution of 1 mg/ml type I collagenase (Gibco, Thermo Fisher Scientific, Waltham, MA, USA) for 60 minutes in a shaking bath at 37°C. The digested pulp tissue was centrifuged and transferred to a flask containing growth medium comprising DMEM supplemented with 10% fetal bovine serum (FBS, Gibco) and 1% penicillin-streptomycin (Sigma-Aldrich, St. Louis, MO, USA), followed by incubation at 37°C in 5% CO_2_ for 10 days prior to the observation of visible hDPSCs colonies. The medium was changed every 3 days. The hDPSCs at passage 6 were used for further experiments.

### Flow cytometric analysis

The hDPSCs were collected in cold Dulbecco's phosphate-buffered saline (DPBS, Thermo Fisher Scientific) and subsequently incubated in the dark at 4°C for 30 min with FITC-conjugated anti-human CD29, FITC-conjugated anti-human CD44, FITC-conjugated anti-human CD105, FITC-conjugated anti-human CD31, FITC-conjugated anti-human CD34, and FITC-conjugated anti-human CD45 (BD Biosciences, San Jose, CA, USA). The samples were read on a flow cytometer (Beckman Coulter, FC500, FL, USA), and the data were analyzed using FlowJo software (Tree Star, San Carlos, CA, USA).

### Cell proliferation assay

FS0.2 and FS2 were used for cell treatments to evaluate the effect of iRoot FS on hDPSC proliferation. Cells cultured in growth medium was set as the blank control group, while BD0.2 and BD2 were set as positive control groups. The hDPSCs were seeded at a density of 1 × 10^4^ cells per cm^2^ onto 48-well tissue culture polystyrene (TCPS) plates (Corning Inc., Corning, NY, USA), and the medium was refreshed every other day. The hDPSCs were treated with a CCK-8 cell proliferation kit (CCK-8; Dojindo, Kumamoto, Japan) according to the manufacturer’s instructions. Briefly, at 1, 3, and 7 days, CCK-8 solution was applied and the cells were incubated for 1 hour. Subsequently, the solution was transferred into 96-well plates, and the absorbance was measured at 450 nm.

### Migration assay

For the wound-healing assay, hDPSCs were seeded onto a 6-well TCPS plate until confluence. Following 24-hour serum-free starvation, a scratch was made using a 10-μL pipette tip. Cells were washed with DPBS to remove the debris and subsequently treated with BD0.2, BD2, FS0.2, and FS2 supplemented with 1% FBS for 24 hours. At 0 and 24 hours, the samples were observed under a phase-contrast microscope, and 4 random photographs were obtained for cell count analysis.

For the Transwell migration assay, a two-chamber Transwell system (Corning) was used as previously described [27]. Briefly, hDPSCs were suspended in serum-free medium at a concentration of 5 × 10^5^ cells per mL. Subsequently, 100 μL cell of suspension was added into the upper chamber, and 700 μL of serum-free BD0.2, BD2, FS0.2, or FS2 culture medium was added into the lower chamber. At 24 hours, the cells under the membrane were stained with 0.1% crystal violet for 20 minutes. To facilitate counting, the samples were further stained with 4’,6-diamidine-2’-phenylindole dihydrochloride (DAPI) and analyzed in 4 random views for each sample under fluorescence microscopy.

### Alkaline phosphatase (ALP) staining and activity analysis

The hDPSCs were seeded onto 24-well TCPS plates (Corning) at 5 × 10^3^ cells per cm^2^ in growth medium for 24 hours. Blank control cells were subsequently cultured in osteogenic medium comprising growth medium plus 100 nM dexamethasone, 10 mM β-glycerophosphate, and 0.28 mM ascorbic acid (Sigma-Aldrich). BD (positive control) or FS cells were cultured in osteogenic medium supplemented with different concentrations of material eluates (the final concentrations were 0.2 and 2 mg/mL, respectively). At 7 and 14 days, ALP staining and activity assays were conducted to determine osteogenic differentiation, as previously described [28,29]. For ALP staining, a leukocyte alkaline phosphatase kit (Sigma-Aldrich) was used, and ALP-positive cells were stained blue. For ALP activity analysis, an ALP quantification kit (Jiancheng Bioengineering Institute, Jiangsu, China) was used according to the manufacturer’s instructions.

### Alizarin red staining and quantification

Alizarin Red S (ARS) staining (Sigma-Aldrich) was performed to determine hDPSC mineralization. At 21 days in osteogenic medium, the cells were fixed with 4% paraformaldehyde and incubated with 2% (w/v) alizarin red for 5 minutes at room temperature. The samples were washed five times with DPBS and observed using a microscope. Subsequently, the samples were destained using 10% (w/v) cetylpyridinium chloride for 15 minutes at room temperature, and the absorbance was measured at 562 nm, as previously described [29].

### RNA extraction and quantitative real-time reverse-transcriptase polymerase chain reaction (qRT-PCR)

The hDPSCs were seeded onto 6-well TCPS plates (Corning) at 5 × 10^3^ cells per cm^2^ and cultured for up to 14 days in osteogenic medium with or without BD (BD0.2 and BD2) and FS (FS0.2 and FS2) supplementation. At 1, 7 and 14 days, the gene expression of collagen type I (COL1) and osteocalcin (OCN) was determined using SYBR Green qRT-PCR (Takara, Otsu, Japan).

The following primer sequences were used for qRT-PCR:

COL1,5’-CCCGGGTTTCAGAGACAACTTC-3’(forward) 5’-TCCACATGCTTTATTCCAGCAATC-3’(reverse);

OCN,5’-CCCAGGCGCTACCTGTATCAA-3’(forward) 5’-GGTCAGCCAACTCGTCACAGTC-3’ (reverse).

### Statistical analysis

The data were analyzed using one-way analysis of variance (ANOVA), followed by the Student-Newman-Keuls test, and P ≤ 0.05 was considered statistically significant.

## Results

### Immunophenotyping of hDPSCs

**Fig 1:**
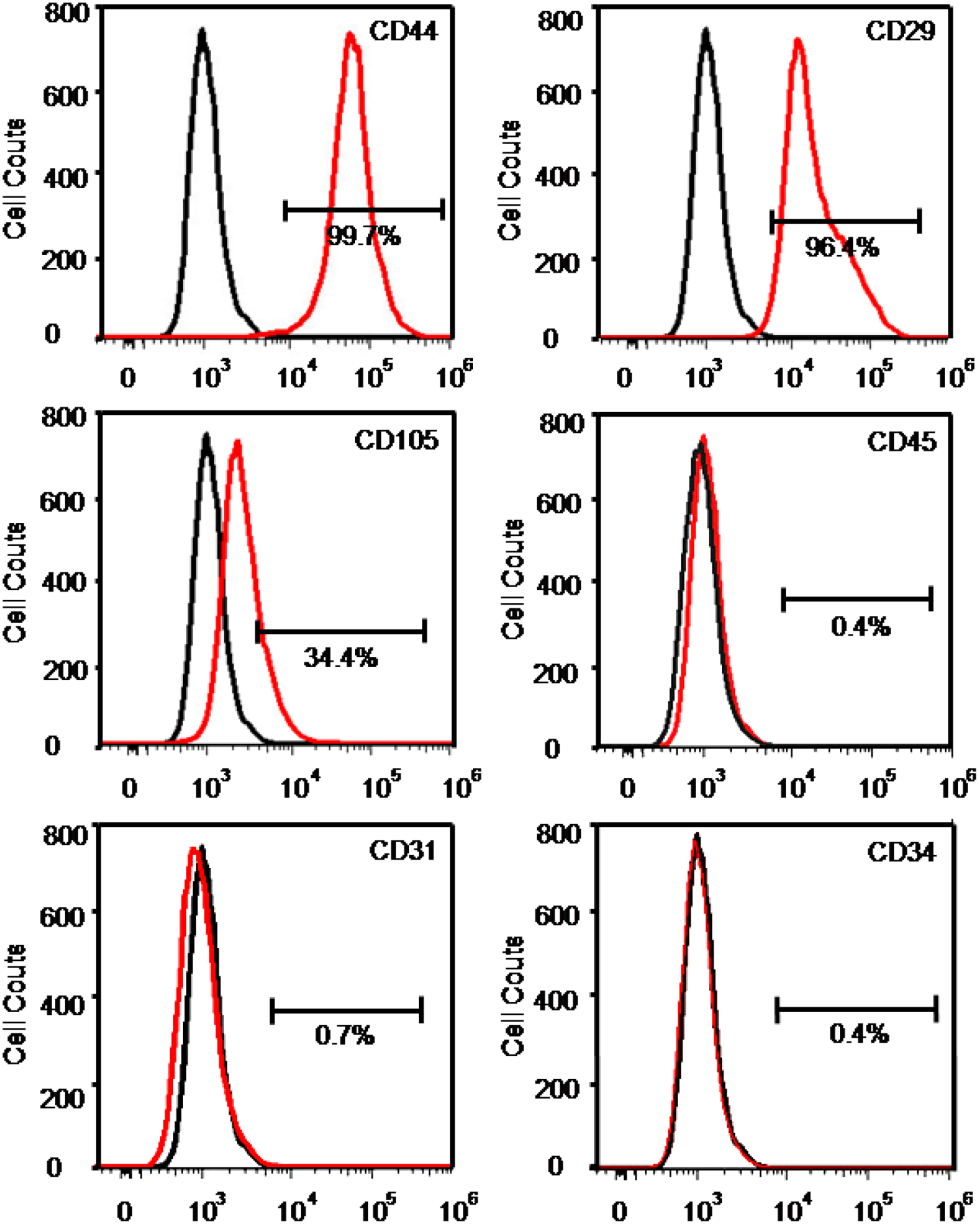
Immunophenotyping of hDPSCs. Flow cytometry showed that hDPSCs highly expressed MSC surface markers and were negative for typical hematopoietic and endothelial cell markers. hDPSCs stained with surface markers are shown as red curves, and control hDPSCs with no staining are shown as black curves.

Mesenchymal stem cell (MSC) surface markers CD29 and CD44 were expressed at levels higher than 96% in hDPSCs (Fig 1). CD105, another MSC surface marker, was expressed at a level greater than 34%. Additionally, the endothelial marker CD31 or the hematopoietic linage markers (CD34 and CD45) were expressed at levels less than 0.8% in hDPSCs. Hence, hDPSCs exhibited consistent and highly typical MSC surface marker expression.

### Cell proliferation

**Fig 2:**
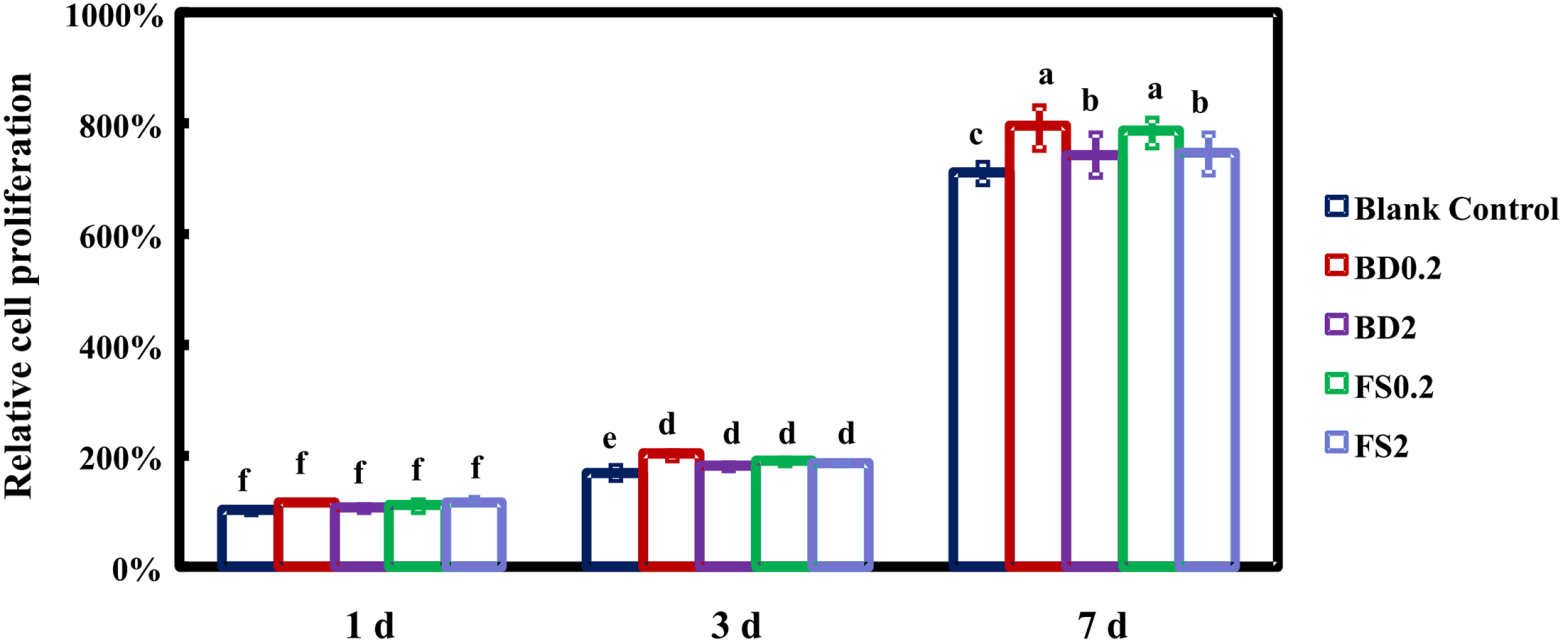
Proliferation of hDPSCs in BD0.2, BD2, FS0.2 and FS2. CCK8 assay was performed after 1, 3 and 7 days (means ± sd; n = 4). Values with dissimilar letters are significantly different from each other (p < 0.05).

An increase in cell proliferation was observed in a time-dependent manner in all groups for the period studied (Fig 2). There was no difference among blank control, BD, and FS groups in cell proliferation at 1 day. The proliferation of the hDPSCs in the FS and BD groups was enhanced compared with that in the blank control group at 3 and 7 days. At 3 days, cell proliferation was similar in BD0.2, BD2, FS0.2, and FS2. However, at 7 days, both FS0.2 and BD0.2 promoted cell growth more significantly than FS2 and BD2. There was no significant difference between FS0.2 and BD0.2 at 7 days.

### Cell migration

**Fig 3:**
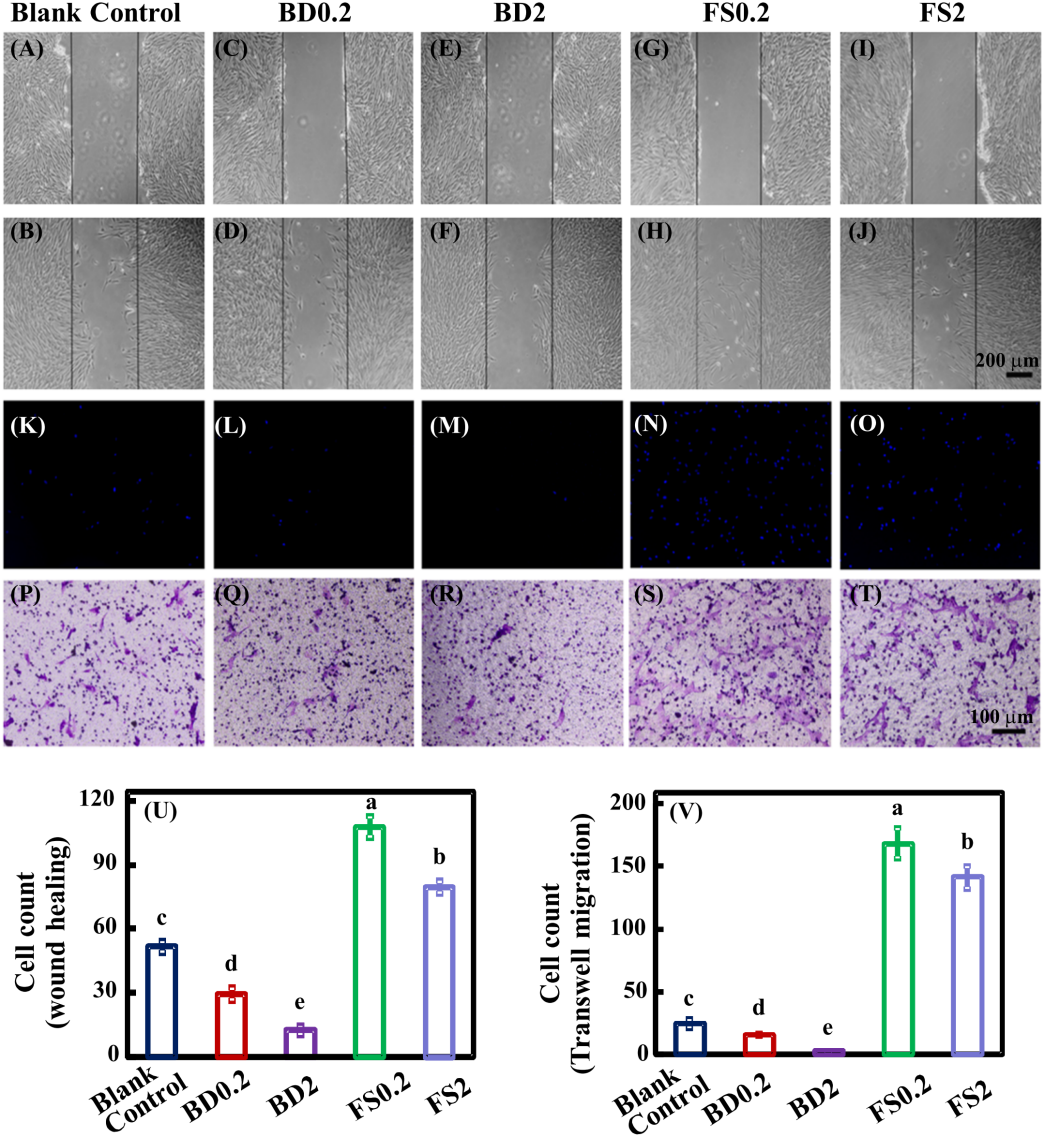
hDPSC migration in blank control group, BD, FS eluates determined using wound healing (A-J) and Transwell (K-T) assays. For the wound-healing assay, photographs at 0 (A, C, E, G, and I) and 24 hours (B, D, F, H, and J) in different groups were obtained for the subsequent evaluation of the number of migrated cells. For the Transwell assay, hDPSCs were added to the upper chamber and incubated for 24 hours, and the migrated cells were stained with both DAPI (K-O) and crystal violet (P-T). Cell migration was quantified using wound healing (U) and transwell (V) assays (means ± sd; n = 4). Values with dissimilar letters are significantly different from each other (p < 0.05).

To explore the effects of FS on human dental pulp stem cell mobility, both wound healing and transwell cell migration assays were used in this experiment. The results showed that in the presence of FS, the migration of hDPSCs was significantly promoted compared with both the blank and BD groups (Fig 3). The hDPSC migration in FS0.2 was more notable than that in FS2. Similarly, the hDPSC migration in BD0.2 was more notable than that in BD2. BD0.2 had no significant effect on hDPSC migration compared with the blank group, while BD2 decreased hDPSC migration.

### Osteogenic differentiation

**Fig 4:**
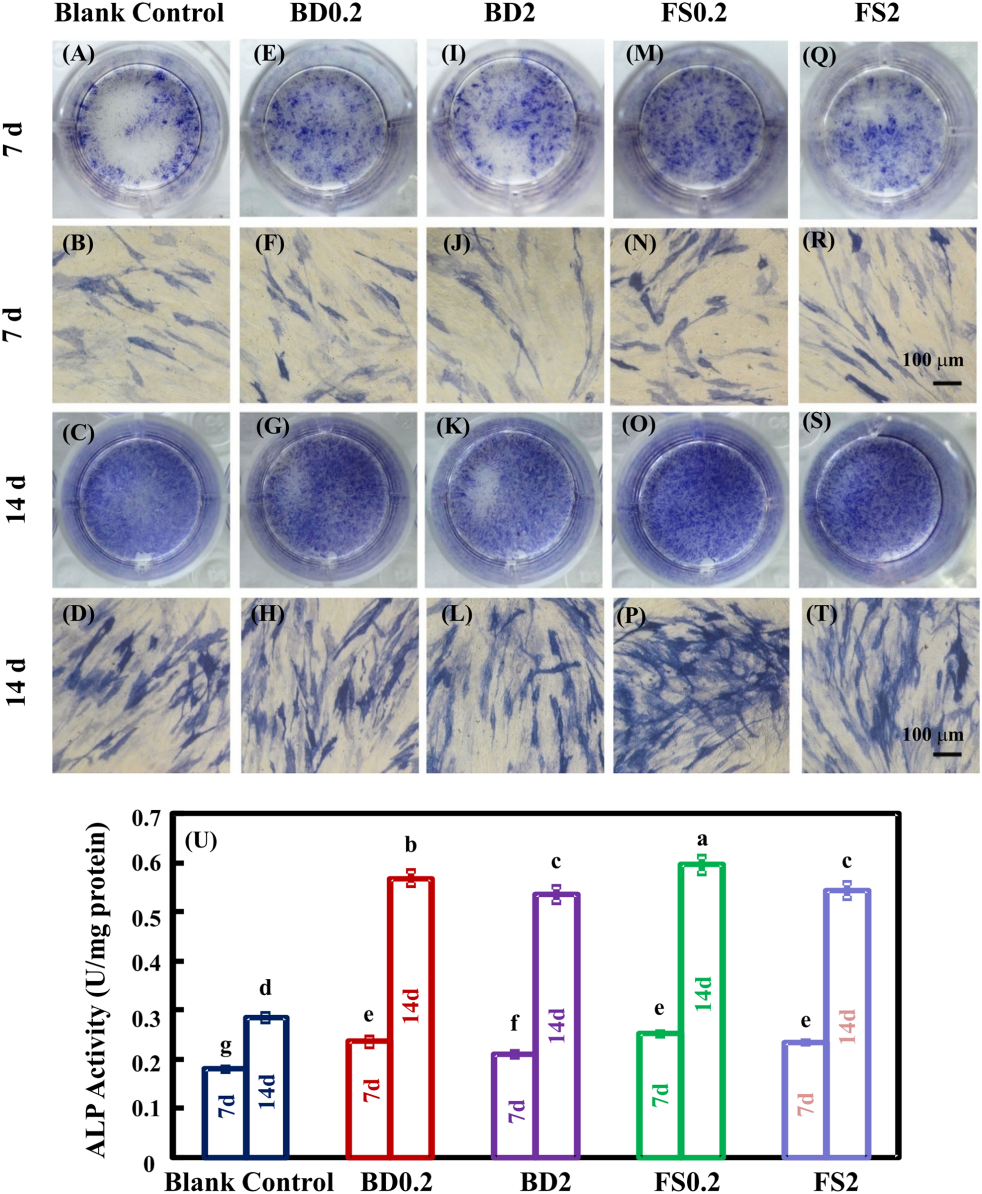
The effect of iRoot FS on ALP activity in hDPSCs. ALP staining of hDPSCs at 7 and 14 days (A-T). Cells cultured in osteogenic medium were used as the blank control group. The osteogenic medium supplemented with BD was used as a positive control. Quantification of ALP activity (means ± sd; n = 4) (U). Values with dissimilar letters are significantly different from each other (p < 0.05).

**Fig 5:**
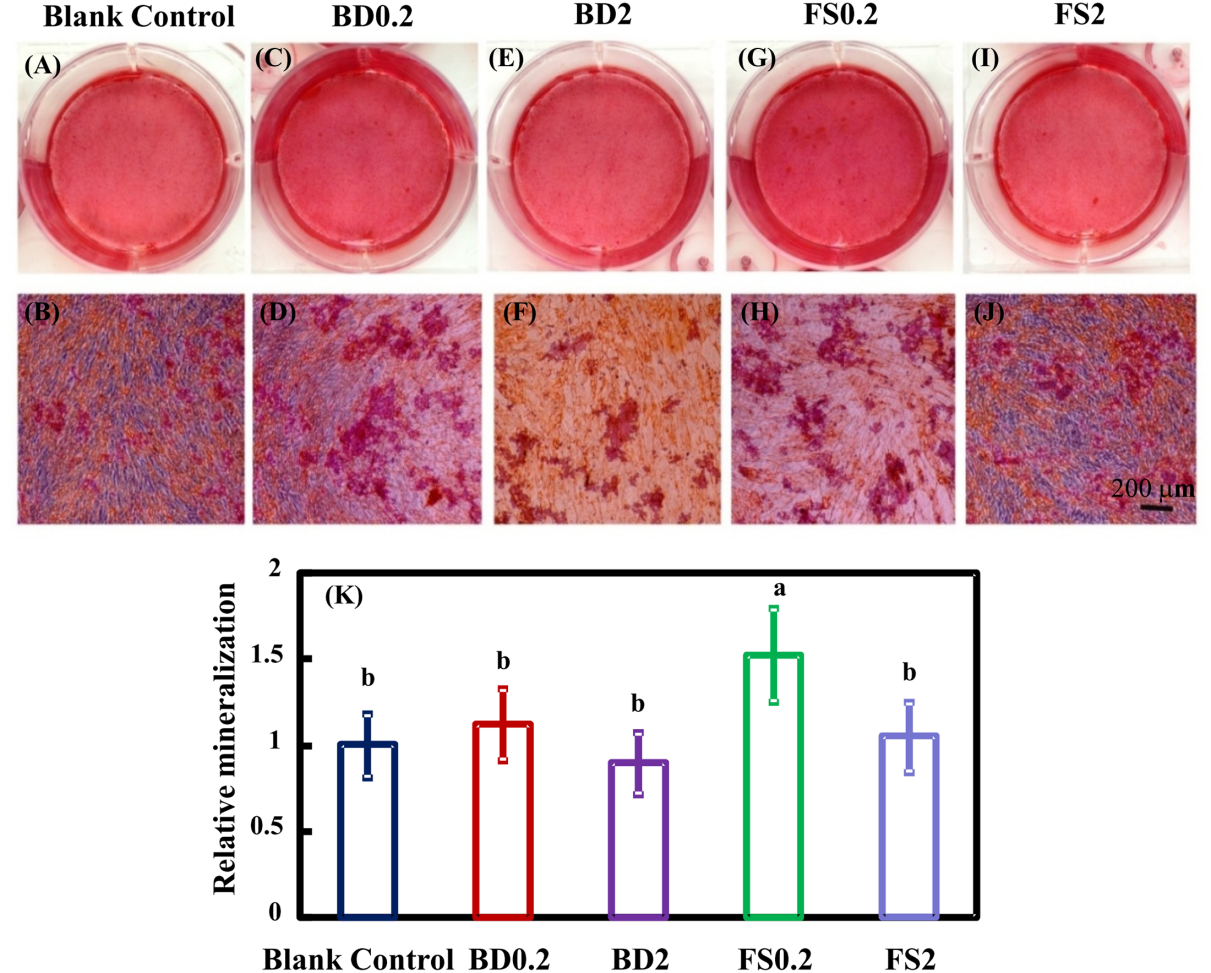
The effects of iRoot FS on mineral synthesis. hDPSCs cultured in osteogenic medium with or without material eluates were stained with Alizarin Red S (ARS) (A-J). Quantitative measurement of ARS staining (means ± sd; n = 4) (H). Values with dissimilar letters are significantly different from each other (p < 0.05).

**Fig 6:**
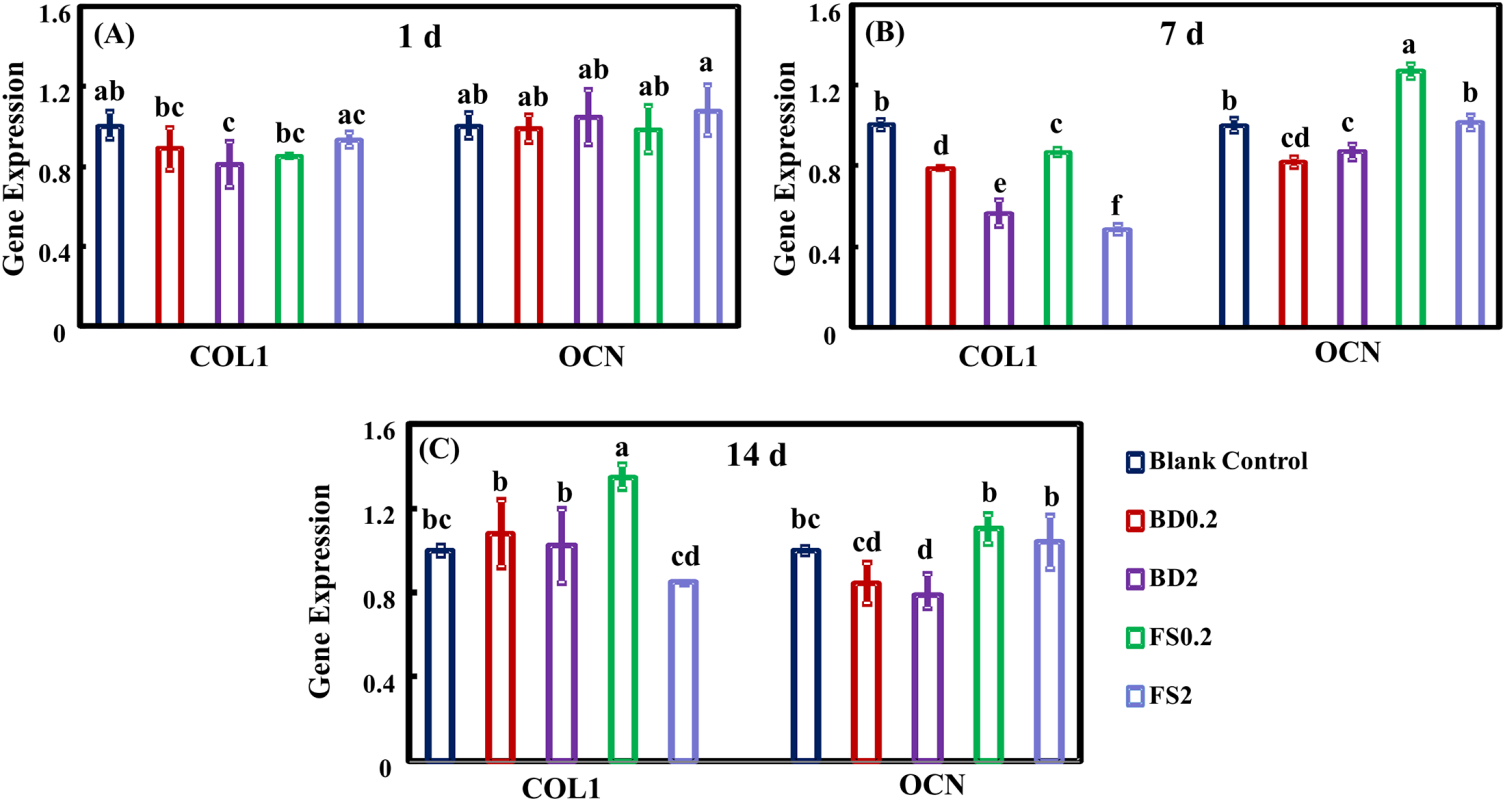
The effect of iRoot FS on osteogenic gene expression in hDPSCs. COL1 and OCN were assayed using qRT-PCR at 1, 7 and 14 days (A-C). Quantification of gene expression (means ± sd; n = 4) (U). Values with dissimilar letters are significantly different from each other (p < 0.05).

The hDPSC differentiation was assessed using an ALP activity assay (Fig 4), ARS staining (Fig 5) and qRT-PCR for osteogenic gene expression (Fig 6). FS and BD displayed higher ALP activity than the blank control group after 7 days. Additionally, a slightly increase in ALP activity was detected in BD0.2, FS0.2, and FS2 compared with BD2. FS0.2 indicated significantly higher ALP activity compared with the other groups at 14 days. In addition, mineral synthesis determined at 21 days was significantly enhanced by FS0.2 but not FS2, BD0.2, and BD2 compared with the blank control group. Notably, FS0.2 dramatically increased the expression of COL1 at 14 days and OCN at 7 days. These results demonstrated that FS0.2 promoted the osteogenic differentiation of hDPSCs.

## Discussion

hDPSCs are a cluster of cells, isolated from dental pulp tissue, which possess stem cell properties with self-renewal ability and multilineage differentiation potential. hDPSCs play pivotal roles in stem cell and regenerative therapies [30–33]. Since Gronthos first isolated and cultured hDPSCs in 2000 [34], these cells have been widely studied for their potential clinical application and easy accessibility. In a recent study, the transplantation of hDPSCs to pulpectomized teeth was authenticated as safe and efficacious for pulp regeneration [35]. Indeed, hDPSCs express osteogenic and angiogenic genes *in vitro* and subsequently fabricate vascularized woven bone *in vivo* [36]. Moreover, according to Giuliani [37], hDPSCs engrafted into teeth extraction wounds trigger compact bone formation. Thus, hDPSCs herald a new dawn for tissue regeneration. Coincidently, pulp-capping therapy is an hDPSC-mediated regenerative process. Hence, hDPSCs are an ideal subject for the present study.

iRoot FS is a pre-mixed, fast-setting bioceramic paste recently introduced for dental use. The present study is the first to investigate the effect of iRoot FS on hDPSCs, the cells that directly or indirectly contact the materials used in capping. The proliferation, migration and differentiation of hDPSCs are indispensable in pulp capping. The results of the present study demonstrated that iRoot FS promotes the proliferation, migration and osteogenic differentiation of hDPSCs. Therefore, iRoot FS is a promising material with favorable biocompatibility for pulp capping.

Notably, in pulp capping, not all cells in the pulp directly contact the material used for capping. Thus, many studies used eluates to evaluate the biological properties of pulp-capping materials [25,27,38,39]. Additionally, material eluates are convenient for observation and easy for quantitative analysis. Hakki *et al*. [38] prepared a concentration gradient of eluates of MTA to study the effect on cell proliferation and observed that concentrations of MTA below 20 mg/mL were nontoxic. Zhao *et al*. [25] showed that MTA at 0.1–2 mg/mL significantly enhanced cell viability. Luo *et al*. [27] suggested that BD, at 0.2 and 2 mg/mL concentrations, promotes cell proliferation. Based on the results of previous studies and preliminary data, we conducted experiments using concentrations of 2 and 0.2 mg/mL.

For direct pulp capping, doctors place the fresh premixed materials on the exposed pulp until the materials solidify. An ideal dental repair material should be biocompatible and capable of promoting cell proliferation. However, the solidified materials might release toxic elements to the surrounding tissues [40]. Therefore, evaluating the effect of the pulp-capping material on cell viability is an important part of material biocompatibility assessment. In the present study, the lower concentrations of FS and BD (FS0.2, BD0.2) showed superiority in promoting cell proliferation. The results for BD were consistent with those of Luo *et al*. [27] and likely attributed to the appropriate release of Ca^2+^ in FS0.2 or BD0.2, which could activate mitochondrial matrix dehydrogenases [41]. Nevertheless, too much Ca^2+^ could inhibit cell proliferation and even cause cell apoptosis [42]. In addition, Zhu *et al*. [43] reported that the eluates of iRoot BP Plus and MTA contained silicon. Previous studies have shown that the high-silicon content of bioceramic materials facilitates cell adhesion and proliferation. Therefore, FS exposed in culture medium could release abundant silicon ions to boost cell bioactivity (Fig 2) [44,45].

Wound healing processes are primarily divided into three stages: the inflammatory and fibrogenic stage, the regenerative stage, and the remodeling stage [27]. Progenitor cell proliferation and migration play essential roles in dentin repair. Calcium silicon-based materials display significant repair capabilities. MTA promotes the proliferation, migration and differentiation of hMSCs [46]. Indeed, MTA-capped teeth demonstrated a higher success rate compared with calcium hydroxide-capped teeth [47]. A rat pulp injury model was established [48], and pulp was capped with ProRoot MTA, Biodentine, or BioAggregate. The results of micro-CT and immunohistochemistry analyses have suggested that Biodentine and BioAggregate promote dentin bridge formation and could be used as alternatives to ProRoot MTA. Shi *et al*. [49] observed that iRoot BP Plus and MTA had similar favorable effects when used in pulp capping. iRoot SP and iRoot FS also demonstrated good cell compatibility [16,22]. The present study is the first to report hDPSC responses to iRoot FS *in vitro* as pulp-capping material.

A fairly common mode of migration involves actomyosin cytoskeleton-mediated changes in the cell body shape [50]. Cell migration could be altered by material-based intervention, co-regulated through physical and chemical mechanisms. MTA could facilitate cell migration [46]. In the present study, hDPSCs in the FS group showed enhanced migration, consistent with Zhang *et al*. [51]. These authors showed that iRoot BP plus could promote dental pulp cell migration through the regulation of FGFR-mediated signaling pathways, which consequently upregulate the expression of focal adhesion molecules and are involved in stress fiber assembly. A previous study reported that low calcium and high phosphoinositide concentrations facilitate cell membrane extension. The ion levels may somehow be associated with the low number of migrated cells in the BD2 group [52,53].

ALP is a protein involved in both early osteogenic differentiation and hard tissue mineralization. In the early stages of mineralization, ALP expression is upregulated to produce inorganic phosphate for hydroxyapatite synthesis [54]. Alizarin red chelates with calcium salt to form an orange-red complex used to detect calcium deposits. The calcium deposits indicate late stage osteogenic differentiation [55]. Calcium silicon-based materials had the potential to boost osteogenic differentiation by increasing ALP expression and calcium deposits [56]. The results of the present study showed that FS0.2 significantly enhanced mineralization, while FS2, BD0.2 and BD2 did not. In the present study, BD increased ALP activity at 7 and 14 days but had no significant effect on hDPSC mineralization at 21 days. These results were not completely consistent with those of previous studies on BD [28], which indicated that BD might increase not only ALP activity but also cellular mineralization at 14 days. This difference may reflect the different time periods for osteogenic differentiation between these studies. In the present study, a period of 21 days was used because a previous study demonstrated significant calcium accumulation from 14 to 21 days during the osteogenesis of MSCs [29]. The COL1 gene encodes the collagenous protein that provides the framework for inorganic deposition, whereas OCN is the major noncollagenous marker of mineralization [57]. The results of the present study showed that FS0.2 initially promoted the expression of OCN and subsequently upregulated COL1 expression. Previous evidence confirmed that initial mineralization may occur without collagen [58]. The specific markers of odontoblastic differentiation, including dentin sialophosphoprotein (DSPP) and dentin matrix protein 1 (DMP1), were also examined using reverse transcription polymerase chain reaction (RT-PCR) and immunofluorescence staining. However, DSPP and DMP1 expression was either not detected in most situations or highly unstable in other situations.

## Conclusions

iRoot FS is a fast setting biomaterial capable of enhancing the proliferation, migration and osteogenic differentiation of hDPSCs. Compared with Biodentine, iRoot FS had comparable ability to promote cell proliferation and performed better in enhancing the migration and mineralization of hDPSCs. Thus, the results of the present study suggest that iRoot FS is a promising bioactive material for pulp capping.
